# A novel scale for triage assessment of frailty in the emergency department (ED-FraS): a prospective videotaped study

**DOI:** 10.1186/s12877-024-04724-9

**Published:** 2024-02-06

**Authors:** Chiat Qiao Liew, Yun Chang Chen, Chih-Wei Sung, Chia-Hsin Ko, Nai-Wen Ku, Chien-Hua Huang, Ming-Tai Cheng, Chu-Lin Tsai

**Affiliations:** 1 Department of Emergency Medicine, National Taiwan University Hospital, Taipei, Taiwan; 2 Department of Emergency Medicine, College of Medicine, National Taiwan University, Taipei, Taiwan; 3 Department of Emergency Medicine, National Taiwan University Hospital Hsin-Chu Branch, Hsinchu, Taiwan; 4 Department of Emergency Medicine, National Taiwan University Hospital Yun-Lin Branch, Hsinchu, Taiwan; 5 Lawrence S. Bloomberg Faculty of Nursing, University of Toronto, Toronto, Canada

**Keywords:** Frailty, Triage, Emergency department

## Abstract

**Background:**

Rapid recognition of frailty in older patients in the ED is an important first step toward better geriatric care in the ED. We aimed to develop and validate a novel frailty assessment scale at ED triage, the Emergency Department Frailty Scale (ED-FraS).

**Methods:**

We conducted a prospective cohort study enrolling adult patients aged 65 years or older who visited the ED at an academic medical center. The entire triage process was recorded, and triage data were collected, including the Taiwan Triage and Acuity Scale (TTAS). Five physician raters provided ED-FraS levels after reviewing videos. A modified TTAS (mTTAS) incorporating ED-FraS was also created. The primary outcome was hospital admission following the ED visit, and secondary outcomes included the ED length of stay (EDLOS) and total ED visit charges.

**Results:**

A total of 256 patients were included. Twenty-seven percent of the patients were frail according to the ED-FraS. The majority of ED-FraS was level 2 (57%), while the majority of TTAS was level 3 (81%). There was a weak agreement between the ED-FraS and TTAS (kappa coefficient of 0.02). The hospital admission rate and charge were highest at ED-FraS level 5 (severely frail), whereas the EDLOS was longest at level 4 (moderately frail). The area under the Receiver Operating Characteristic curve (AUROC) in predicting hospital admission for the TTAS, ED-FraS, and mTTAS were 0.57, 0.62, and 0.63, respectively. The ED-FraS explained more variation in EDLOS (R^2^ = 0.096) compared with the other two methods.

**Conclusions:**

The ED-Fras tool is a simple and valid screening tool for identifying frail older adults in the ED. It also can complement and enhance ED triage systems. Further research is needed to test its real-time use at ED triage internationally.

**Supplementary Information:**

The online version contains supplementary material available at 10.1186/s12877-024-04724-9.

## Introduction

In 2022, the global population of individuals aged 65 years or older was 771 million, with a projected increase to 1.6 billion by 2050 [1]. The proportion of this age group in the global population was 9.7% in 2022, with a projected rise to 16.4% by 2050 [1]. The aging population often faces challenges in accessing appropriate and timely care [2–4]. This can lead to emergency department (ED) utilization, especially for those with limited access to primary care or specialty services [2–4]. The increase in demand for ED services due to an aging population creates challenges for healthcare systems, including longer length of stay, increasing hospitalization, overcrowding and longer wait times [2, 3, 5]. As a result, emergency care geared toward the geriatric population has become increasingly important.

Frailty is a common condition in older adults and is associated with increased vulnerability to adverse outcomes [6–10]. Frailty in acute care settings is characterized by a rapid decline in physical, cognitive, or social functioning [6, 7, 10]. The ED is a crucial point of care for older adults with acute medical issues, and there is growing interest in measuring frailty in the ED setting. The timely recognition of frail patients in the ED helps with clinical decision-making because frailty predicts a range of adverse health outcomes. This identification also may help initiate frailty pathways comprising hospital geriatric units, providing integrated and patient-centered care. Though there are frailty screening tools, such as the Cardiovascular Health Study (CHS) [6] scale and the Rockwood Clinical Frailty Scale (CFS) [11], it is challenging to rapidly identify frail persons in a hectic ED [12–14]. Some frailty screening tools may be time-consuming to administer, which also creates a burden for ED staff and limits the feasibility of universal screening in the ED. Frailty screening tools often do not consider both acute and chronic conditions, both of which may contribute to acute exacerbations of chronic conditions commonly seen in the ED [15, 16]. As such, frailty screening tools developed for community-dwelling older adults may not be as relevant or useful in the ED [15, 16]. There is no research on quick, observation-based frailty assessment at the ED triage that we are aware of. In our prior study, we developed and validated an observation-based (physician gestalt) triage method that performed similarly to computer algorithm-based triage [17]. This physician gestalt-based quick assessment may also be employed to assess frailty at ED triage to augment existing triage systems.

In this study, we aimed to develop and validate a novel triage assessment of frailty in the emergency department. This novel ED-specific, observation-based frailty scale was developed via physicians’ review of video recordings of triage processes. This frailty tool was then validated against hospitalization, ED length of stay (EDLOS), and ED charges. Frailty assessed by this tool was also added to the existing triage system for predicting the aforementioned ED outcomes.

## Methods

### Study design, setting, and population

This was a geriatric sub-study of a prospective observational study [17] that was conducted in the ED of the National Taiwan University Hospital (NTUH) from May 2020 to March 2022. The NTUH is a tertiary academic medical center with approximately 2,400 beds and 100,000 ED visits annually. Patients presenting to the ED were prospectively enrolled by trained research personnel following a standardized protocol. Inclusion criteria required that the patient be aged 20 years or older (legal age for adults in Taiwan) and able to provide informed consent. Patients were excluded if they exhibited consciousness disturbance (coma or intoxication), required isolation for infection control, or needed immediate cardiopulmonary resuscitation. Patients with cognitive impairment were still included, and consent was obtained from their legally authorized representatives or next of kins. The triage process was documented using a high-sensitivity camera and a clip-on Bluetooth microphone, capturing patients' facial images and conversations between patients, their families or companions, and the triage nurses. Consecutive patients during an enrollment shift were approached. This study was approved by the NTUH Institutional Review Board, and informed consent was obtained from all participants or their surrogates. This study focused on a subset of the main study's geriatric population, namely patients aged 65 years or older.

### Measurements

In this study, we developed the Emergency Department Frailty Scale (ED-FraS), a new scale for frailty assessment tailored to the unique environment of the ED. Given the differences between ED settings and outpatient clinics, ED frailty assessments are preferable to be conducted in a relatively short period of time and at the first patient encounter in the ED (i.e., triage area). We developed the ED-FraS considering commonly used frailty scales for older adults, such as the CHS scale,11 the CFS,19 and the Stable gait, Unstable gait, Help needed to walk, Bedridden (SUHB) scale.5 After multiple rounds of research meetings and discussions, the research team reached a consensus on the definition and scoring criteria of the ED-FraS (Table 1).

**Table 1:** The Emergency Department Frailty Scale (ED-FraS)

	Level	Description
Level 1	1 Healthy	Individuals exhibiting positive well-being and agility, with no apparent constraints on their range of motion
Level 2	2 Chronic Conditions	Individuals with well-managed chronic conditions (e.g., diabetes mellitus or hypertension) may exhibit slightly reduced mobility, yet no significant limitations in their range of motion are observed
Level 3	3 Mildly Frail	Individuals with noticeably sluggish or limited body movement may require assistive devices but not the help of others. They are able to walk independently, occasionally needing a hand-held walker for support. Their cognitive function and reaction times may be observed as delayed at times
Level 4	4 Moderately Frail	Individuals requiring assistance for mobility and unable to walk independently. They are characterized by delayed cognitive function and reaction times, which may be inefficient and time-consuming, yet they remain capable of communication. This category typically includes those who need a wheelchair for transfers (e.g., entering the emergency department), and/or require a dedicated caregiver for mobility assistance
Level 5	5 Severely Frail	Individuals who are completely dependent on others for mobility (e.g., bedridden) or unable to communicate due to cognitive impairment

When evaluating a patient with varying degrees of impairment across different dimensions (e.g., mobility and cognition dimensions), the assessment should be based on the most severe aspect of the patient's condition

The ED-FraS comprises five distinct levels for measurement. This scale was designed to be completed along with a typical triage process and was based primarily on observation and review of triage note. The patient's frailty was assessed using the ED-FraS and was categorized into five levels, ranging from healthy (level 1), chronic conditions (level 2), mildly frail (level 3), moderately frail (level 4), and severely frail (level 5).

The Taiwan Triage and Acuity Scale (TTAS) [18], a computerized triage software adapted from the Canadian Triage and Acuity Scale (CTAS), has been used for ED triage in Taiwan since 2010. The TTAS classifies patients into five levels, ranging from resuscitation (level 1), emergent (level 2), urgent (level 3), less urgent (level 4), to non-urgent (level 5).

To incorporate the ED-FraS into the currently used TTAS, the modified TTAS (mTTAS) was created. The mTTAS was a modification to the TTAS for older patients who were considered frail. If a patient's ED-FraS level was 3 or above (mildly, moderately, or severely frail), his/her TTAS level was tuned up by one level, with level 1 being the upper limit. The cutoff point of 3 (mildly frail) in the ED-FraS was selected a priori to define frailty. Similar to the CFS, a cutoff point of 5 (living with mild frailty) was used in most studies to define frailty [11, 13, 19].

The triage process begins with an open question regarding the chief complaint of each patient. Data were collected, including triage date and time, levels of consciousness, demographics, pre-existing comorbidities, structured chief complaints, and vital signs (body temperature, heart rate, respiratory rate, systolic and diastolic blood pressure, oxygen saturation).

### Video review and ED-FraS scoring

Before scoring the ED-FraS, the video records (*n* = 1,009) underwent quality checks to ensure optimal image and sound quality. Five emergency physicians were recruited as reviewers and trained through educational meetings. All five emergency physicians involved have completed specialized training in emergency medicine. Among them, two have over ten years of clinical tenure, one has five years, and the remaining two have two years. Our reviewer panel reviewed and discussed online educational videos about frailty and geriatric assessment in general during the educational meetings. Triage electronic health records were provided to the reviewers, with TTAS levels masked. For each enrolled case, reviewers watched the video and read the triage record before classifying the patient into separate ED-FraS levels. The triage records contained only key information, such as chief complaints, vital signs, and past medical history; there was no information regarding patient outcomes. When evaluating a patient with varying degrees of impairment across different dimensions (e.g., mobility and cognition dimensions), we based our assessment on the most severe aspect of the patient's condition. The first ten videos were scored by all reviewers and analyzed as pilot data, while subsequent videos were rated independently. The intraclass correlation coefficient (ICC) between the reviewers reached 0.863 for the pilot data, indicating a high level of inter-reviewer agreement on ED-FraS scoring. Following a pilot testing phase, these physicians then independently rated the video recordings. The videos were lined up according to the recording date/time and were distributed to the five reviewers in a fixed sequential order. This ensured that each physician was assigned different patients. Periodic investigator consensus meetings were held to discuss and resolve any issues related to the ED-FraS scoring.

### Outcomes

The primary outcome of this study was hospital admission following the ED visit. Secondary outcomes included the EDLOS and the total charges associated with the ED visit. The EDLOS was defined as the time elapsed between the patient's arrival at triage and departure from the ED. The total charges (in New Taiwan Dollars, NT$) encompassed registration fees, physician fees, medication charges, and self-pay fees.

### Statistical analysis

Descriptive statistics are presented as proportions (with 95% confidence intervals [CI]), means (with standard deviations [SD]), or medians (with interquartile ranges [IQRs]). Student's t-tests, Chi-square tests, or Mann–Whitney tests were employed to examine bivariate associations as appropriate. The kappa statistic was used to measure the correlation between the two scales, and Spearman's correlation coefficient was also calculated. A weighted kappa statistic was also reported with quadratic weighting. Receiver operating characteristic (ROC) curves for hospital admission using ED-FraS, TTAS, and mTTAS were plotted, with the area under the ROC (AUROC) representing the discriminatory ability of prediction. The DeLong test was used for the comparison between AUROCs. Additionally, linear regression models for EDLOS and charge prediction using ED-FraS, TTAS, and mTTAS were performed, providing R^2^ as the coefficient of determination. Although the EDLOS and charge data were skewed, we did not transform the data because parametric methods are robust to non-normality with sufficiently large samples [20]. Thus, simple linear regression models were used to predict EDLOS and charges. The variation in outcome explained by triage methods was quantified by R^2^, also known as the coefficient of determination. All analyses were conducted using Stata 16.0 software (Stata Corp, College Station, TX). All *P*-values are two-sided, with *P* < 0.05 considered statistically significant.

## Results

The patient selection process for the study is shown in Online Supplementary Figure S1. Of the 1,974 patients approached, 965 were excluded largely due to refusal to participate or ineligibility. Of the 1,009 enrolled patients, 753 were subsequently excluded for various reasons (645 due to age < 65 years, 52 for audio and 19 for video issues, 10 used as pilot data, 9 referred to the outpatient clinic, 9 due to repeat visits, and 9 for restricted access to records). The final analysis included 256 patients.

Table 2 displays the clinical characteristics of the patients. The average age was 75.1 years, with 113 patients (44.1%) being female. Six percent of the patients arrived by ambulance. Ten patients had dementia. The most common chief complaints were abdominal pain (9.4%), chest pain (8.6%), and dizziness (6.3%). The majority of patients were triaged at level 3 (80.9%). The ED-FraS identified 69 patients as frail (level 3 or above, 27%). The majority of the patients were level 2 (with chronic conditions, 57.4%), followed by level 3 (mildly frail, 23.4%). The median triage duration was 2.63 min. The hospital admission rate was 21.5%, and the median EDLOS was 3.1 h.

**Table 2:** Baseline clinical characteristics of emergency department patients

Variable	*N* = 256
Age, mean (SD), yr	75.1 (7.4)
Female sex, n (%)	113 (44.1)
Arrival by ambulance, n (%)	6 (2.3)
Comorbid condition, n (%)	
Hypertension	86 (33.6)
Cardiac disease	76 (29.7)
Diabetes Mellitus	69 (27.0)
Cancer	68 (26.6)
Chronic kidney disease	20 (7.8)
Dementia	10 (3.9)
Stroke	8 (3.1)
Most common chief complaint, n (%)	
Abdominal pain	24 (9.4)
Chest pain	22 (8.6)
Dizziness	16 (6.3)
Skin inflammation/swelling	16 (6.3)
Fever	9 (3.5)
Palpitations	8 (3.1)
Edema	8 (3.1)
Flank pain	8 (3.1)
Dyspnea	7 (2.7)
General weakness	7 (2.7)
Melena	7 (2.7)
Blunt injury, upper extremities	7 (2.7)
Triage level, n (%)	
1 (Resuscitation)	1 (0.4)
2 (Emergent)	36 (14.1)
3 (Urgent)	207 (80.9)
4 (Less urgent)	7 (2.7)
5 (Non-urgent)	5 (2.0)
ED-FraS level, n (%)	
1 (Healthy)	40 (15.6)
2 (Chronic condition)	147 (57.4)
3 (Mildly frail)	60 (23.4)
4 (Moderately frail)	8 (3.1)
5 (Severely frail)	1 (0.4)
Triage duration, median (IQR), min:sec	2:38 (2:07–3:41)
Hospital admission, n (%)	55 (21.5)
ED length of stay, median (IQR), hr	3.1 (1.8–10.5)

Abbreviations: *SD* Standard deviation, *ED-FraS* Emergency department frailty scale, *IQR* Interquartile range, *ED* Emergency department

Table 3 demonstrates the agreement between ED-FraS and TTAS. Most discrepancies occurred at the lower left off-diagonal, where most patients were triaged at level 3 but were not considered frail (either healthy or with chronic conditions). The kappa statistic showed a very weak agreement of 0.02, and so did the weighted kappa statistic (0.09). The Spearman’s correlation coefficient between the TTAS and the ED-FraS was only 0.12 (*p* = 0.057). A bubble graph showed the relationship of the two scales (Online Supplementary Figure S2).

**Table 3:** Agreement between triage level and frailty scale

	Frailty scale					
Triage level	1	2	3	4	5	Total
1	0	0	0	1	0	1
2	5	8	10	2	1	36
3	32	122	48	5	0	207
4	0	6	1	0	0	7
5	3	1	1	0	0	5
Total	40	147	60	8	1	256

Table 4 presents the stratification of study outcomes by the ED-FraS. The hospital admission rates gradually increased with higher frailty levels, with a 100% admission at ED-FraS level 5 (*P* = 0.002). Similar trends were observed with ED charges and EDLOS, with the exception that the longest ED stay was observed at ED-FraS level 4. Further breakdowns of EDLOS by admission status showing that the increase in EDLOS with higher frailty levels was more evident among patients who were discharged from the ED (*p* = 0.03).

**Table 4:** Study outcomes by frailty scale

	Frailty scale					
**Overall**						
Outcome	1 (*n* = 40)	2 (*n* = 147)	3 (*n* = 60)	4 (*n* = 8)	5 (*n* = 1)	*P* value
Admission, n (%)	6 (15.0)	26 (17.7)	19 (31.7)	5 (62.5)	1 (100)	0.002
ED length of stay, median (IQR), hour	2.4 (1.2–5.1)	2.8 (1.7–6.2)	5.2 (2.5–23.8)	45.9 (14.7–86.5)	40.1 (40.1–40.1)	< 0.001
Charge, median (IQR), NT$	2,379.5 (1,613–7,352.5)	3,456 (2,193–6,799)	6,240.5 (3,634–14,653.5)	11,093.5 (10,361.5–19,268.5)	19,206 (19,206–19,206)	< 0.001
***Subgroup: Admitted***						
	1 (*n* = 6)	2 (*n* = 26)	3 (*n* = 19)	4 (*n* = 5)	5 (*n* = 1)	*P* value
ED length of stay, median (IQR), hours	20.4 (11.7–21.6)	23.3 (6.0–43.2)	25.2 (4.2–58.4)	49.5 (42.3–98.4)	40.1 (40.1–40.1)	0.172
***Subgroup: Discharged***						
	1 (*n* = 34)	2 (*n* = 115)	3 (*n* = 39)	4 (*n* = 3)	5 (*n* = 0)	*P* value
ED length of stay, median (IQR), hours	2.2 (0.9–3.8)	2.5 (1.4–3.9)	4.1 (1.9–7.6)	5.6 (1.2–74.6)		0.029

Abbreviations: *ED* Emergency department, *IQR* Interquartile range

**Fig. 1:**
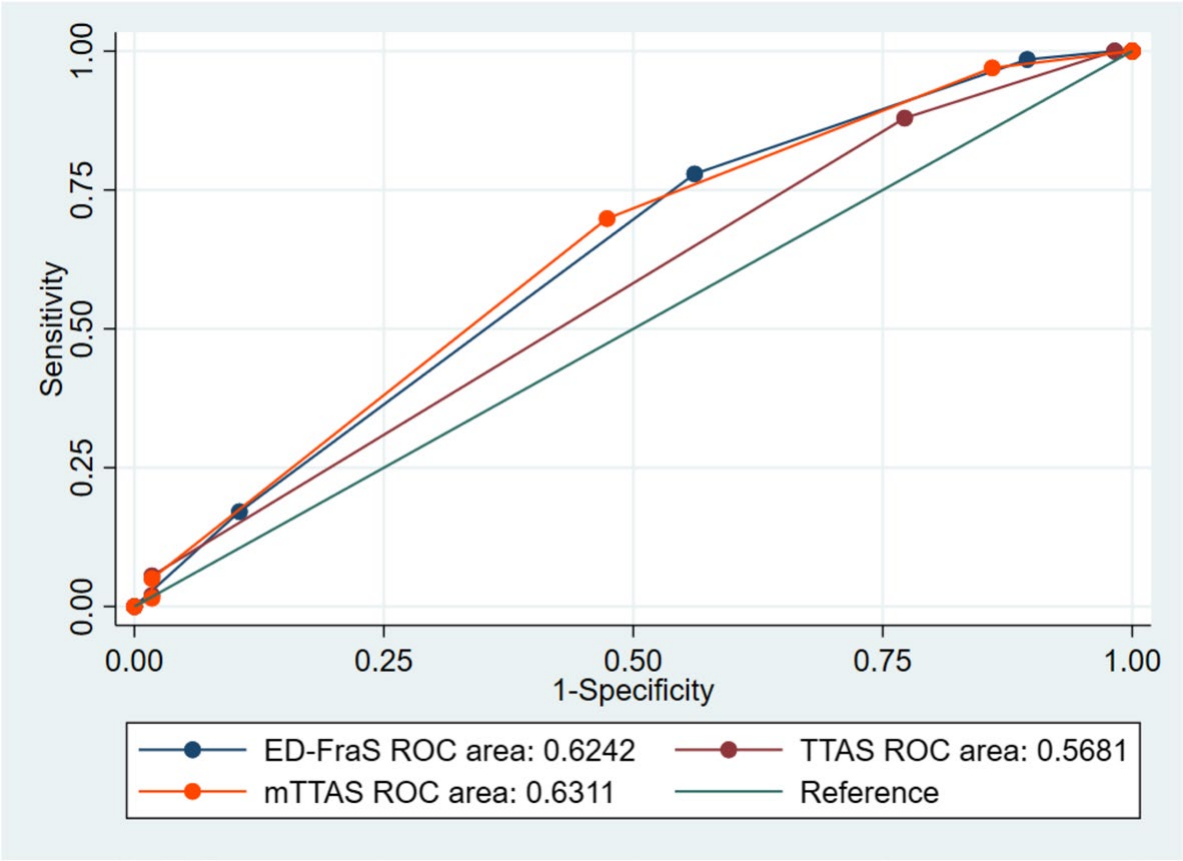
Receiver operating characteristic curves for the three methods. The diagonal line represents a model of no discriminatory ability. Abbreviations: ED-FraS = Emergency Department Frailty Scale (ED-FraS); TTAS = Taiwan Triage and Acuity Scale; mTTAS = modified TTAS

Figure 1 displays the ROC curves for triage, frailty and combined scales. The AUROCs for ED-FraS, TTAS, and mTTAS were 0.62 (95% CI: 0.55–0.70), 0.57 (95% CI: 0.51–0.63), and 0.63 (95% CI: 0.56–0.71), respectively. No statistically significant difference was found across the AUROCs (*p* = 0.07). Of note, compared with the original TTAS, the mTTAS had a superior discriminatory ability for hospital admission (*p* = 0.03).

For EDLOS, the linear regression models showed a slightly higher coefficient of determination of the ED-FraS (R^2^ = 0.096), compared with that of the TTAS (R^2^ = 0.043) and mTTAS (R^2^ = 0.081). The ED-FraS was positively associated with the EDLOS (beta-coefficient, 8.9 h per 1-level increase, *p* < 0.001), while the other two triage scales were negatively associated with the EDLOS (*p* = 0.001 for TTAS, *p* < 0.001 for mTTAS).

For ED charges, it was the mTTAS (R^2^ = 0.092) that explained more variance than the ED-FraS (R^2^ = 0.065) and the TTAS (R^2^ = 0.064). The ED-FraS was positively associated with the ED charges (beta-coefficient, NT$3,469 per 1-level increase, *p* < 0.001), while the other two triage scales were negatively associated with the ED charges (*p* < 0.001 for both).

## Discussion

In this prospective videotaped study, we developed and validated a novel frailty assessment tool in the ED. This simple five-level ED-FraS is designed to be completed at ED triage within minutes with observation and triage information, considering both acute and chronic conditions. For predictive validity, increased ED-FraS levels were associated with increased hospitalization rates, longer EDLOS, and higher ED charges. In addition, the ED-FraS appeared to measure a different dimension than what triage acuity intends to measure, as evidenced by low kappa and correlation coefficients (divergent validity). Compared with the traditional triage tool (TTAS), the ED-FraS or its augmented version of the triage tool (mTTAS) performed better in predicting hospital admission, EDLOS, and ED charges.

This prospective study was, to the best of our knowledge, the first videotaped evaluation for frailty assessment at ED triage. The ED-FraS is primarily an observational assessment and therefore is well suited to ED triage where the patient's physical appearance, mobility, cognitive function, and overall clinical presentation can be assessed all at once. Because ED triage is where the first patient encounter occurs, this brief assessment can also be incorporated into the screening, brief intervention and referral to treatment (SBIRT) model. For example, for those who screen positive for the ED-FraS (level 3 or above), a patient-tailored care plan can be developed to include the patient's goals and preferences and follows the patient after ED discharge. The ED-FraS also has predictive validity as ED outcomes (e.g., admission rates) are associated with ED-Fras frailty levels. This finding is consistent with existing literature on frailty and adverse outcomes in older patients [21–25]. In other words, the ED-FraS demonstrated fair discrimination in identifying older adults with frailty who were at high risk of adverse outcomes. Although there are some frailty screening tools used in EDs, including CHS and CFS, they were primarily used in the inpatient or the community setting. In addition, based on our experience during the development process, the ED-Fras was easy to use and required minimal time to complete, which is particularly important in the fast-paced environment of the ED.

The ED-FraS we developed is different from other frailty tools in that it encompasses both acute and chronic conditions. As such, our ED-specific tool can be used to predict short-term ED outcomes, as opposed to post-ED longer-term outcomes that traditional frailty tools aim to predict. For example, in a UK study, emergency physicians found the CFS scores useful at ED triage for predicting post-ED outcomes [26]. Because the ED-FraS considers chronic conditions, it can also be incorporated into triage acuity tool just as other traditional frailty scales. The results of a large cohort study conducted in Canada revealed that frailty and triage acuity are distinct but complementary measures in the ED [27]. The findings suggest that EDs may benefit from implementing comprehensive frailty screening as a follow-up to triage assessments [27]. A study conducted in Spain has introduced an ED triage tool that incorporates frailty screening into their triage system to identify at-risk or frail older patients [28]. The TTAS was adapted from the CTAS and has been widely used in every EDs in Taiwan [18]. Despite the overall validity and reliability of the TTAS, it lacks a modifier for older patients [29, 30]. Ng et al. have reported that the incorporation of the CFS into TTAS has the potential to reduce the under-triage of older adults in the ED [31]. Similarly, the mTTAS, which incorporated the frailty screening tool we developed, showed improved ability in predicting all ED outcomes.

In terms of performance of predicting hospitalization, compared with other frailty tools used in the ED, the ED-FraS and mTTAS demonstrated similar AUROCs. For example, the AUROC for predicting hospitalization using the CFS at ED triage was 0.67 (95% CI, 0.66 to 0.68) [32]. The other two frailty screening tools in the ED include the Identification of Seniors at Risk (ISAR) tool and the Triage Risk Screening Tool (TRST). The ISAR can also be used to identify elderly individuals at high risk of adverse events, with an AUROC for predicting hospital admissions of 0.65 (95% CI: 0.62–0.68) [33]. The TRST, consisting of items assessing functional status, recent hospitalizations, cognitive impairments, and polypharmacy, achieved AUROC values of 0.66 for admission prediction [34]. Overall, the ED-FraS demonstrated comparable performance to existing frailty screening tools in terms of predicting short-term ED outcomes.

One notable strength of the study was the use of video recordings of the triage process, which allowed for a more comprehensive evaluation of the patients' conditions and improved the accuracy of the frailty assessment. A picture is worth a thousand words; the gestalt methods we developed previously for triage assessment may also be applied to frailty assessment, especially at ED triage, where senior nurses conducted interviews. Additionally, the high inter-reviewer agreement on ED-FraS scoring indicated the scale's reliability. This high agreement serves as the rationale for the training of more healthcare providers and wider adoption of the scale in the ED.

The study has several limitations. Firstly, the study did not document the follow-up outcomes of these patients beyond their ED visits. Therefore, it remains unknown whether frail older patients identified by the ED-FraS are associated with mortality on the ward or ED revisits. Secondly, it was only validated in a single center, which may limit its generalizability to other EDs. Specifically, the TTAS is only used in Taiwan. Clinicians in other countries will need to devise a way to integrate frailty into their own triage system. Thirdly, the study did not include many severely ill patients as these patients were not easy to recruit prior to treatment at the ED triage. This could affect the accuracy and reliability of the ED-FraS or mTTAS among these high-acuity patients. Although only essential information with masked TTAS levels was provided to the reviewer, there remains a possibility of unblinding (i.e., using alternative sources to determine frailty scores). Lastly, the ED-FraS needs to be implemented by triage nurses to test its real-world effectiveness. Future refinements are also needed to improve its modest discriminatory performance. Replicating this study with triage nurses or nursing staff in the ED is crucial to observing the tool's performance across various disciplines and settings.

## Conclusions

In conclusion, we developed and validated a novel ED-Fras tool that is simple and easy to administer as a screening tool for identifying frail older adults in the ED. Its straightforward design and proven validity and reliability make it a promising frailty assessment tool in the ED. The ED-FraS can also complement and enhance current triage systems. Further research is needed to test its real-time use at ED triage.

### Supplementary Information

**Additional file 1.**

**Additional file 2.**

## Data Availability

The datasets used and/or analyzed during the current study are available from the corresponding author on reasonable request.

## Abbreviations

ED: Emergency department
ED-FraS: Emergency Department Frailty Scale
TTAS: Taiwan Triage and Acuity Scale
mTTAS: Modified Taiwan Triage and Acuity Scale
EDLOS: ED length of stay.
AUROC: Area under the Receiver Operating Characteristic curve
CHS: Cardiovascular Health Study
CFS: Rockwood Clinical Frailty Scale
SUHB: Stable gait, Unstable gait, Help needed to walk, Bedridden.
CTAS: Canadian Triage and Acuity Scale
ICC: Intraclass correlation coefficient
CI: Confidence intervals
SD: Standard deviations
IQR: Interquartile ranges
ROC: Receiver operating characteristic
SBIRT: Screening, brief intervention and referral to treatment.
ISAR: Identification of Seniors at Risk tool
TRST: Triage Risk Screening Tool
