# Supplementary figures and images for: A novel scale for triage assessment of frailty in the emergency department (ED-FraS): a prospective videotaped study
Source: BMC Geriatr. 2024 Feb 6;24:137. doi: 10.1186/s12877-024-04724-9 (PMC10848459; doi:10.1186/s12877-024-04724-9)

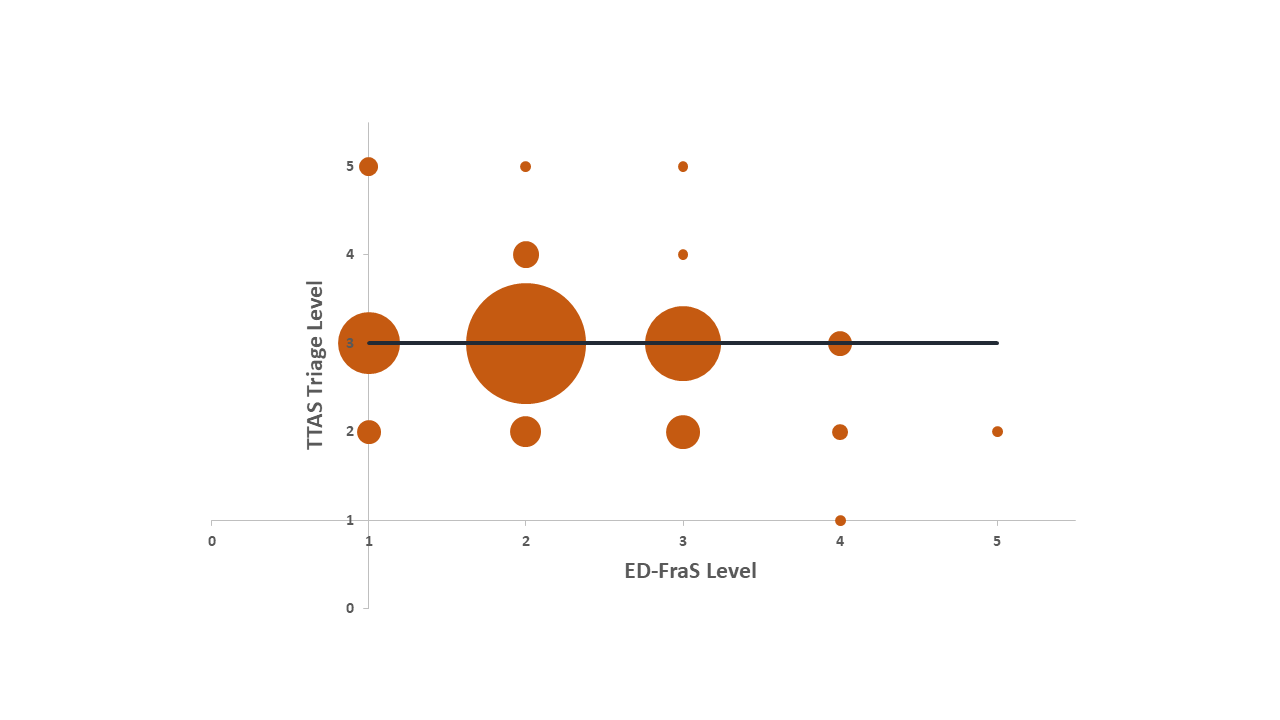

Supplement: Supplementary file 2 — Additional file 2. [file 12877_2024_4724_MOESM2_ESM.tif]
